# Antispasmodic and antidiarrhoeal activity of the fruit of *Rosa moschata* (J)

**DOI:** 10.1186/1472-6882-14-485

**Published:** 2014-12-13

**Authors:** Niaz Ali, Hina Alam, Aslam Khan, Ghayour Ahmed, Wadood Ali Shah, Muhammad Nabi, Muhammad Junaid

**Affiliations:** Department of Pharmacology, Institute of Basic Medical Sciences, Khyber Medical University, Peshawar, Pakistan; Department of Pharmacy, Kohat University of Science and Technology, Kohat, Pakistan; Department of Pharmacy, University of Malakand, Chakdara Dir, Lower, Pakistan

**Keywords:** *Rosa moschata*, Antidiarrhoeal, Antispasmodic, Ca^++^ antagonist, Verapamil

## Abstract

**Background:**

The fruit of *Rosa moschata* has traditionally been used for the treatment of abdominal spasm and diarrhoea. Therefore, the aim of this study was to investigate mechanism(s) responsible for its medicinal use in gut spasm and diarrhea.

**Methods:**

Hydro-methanolic extract of *Rosa moschata* (Rm.Cr) was studied in isolated rabbit’s jejunal preparations for possible antispasmodic activity. Based upon *in vitro* relaxant activity in isolated gut preparations, *in vivo* antidiarrheal activity was carried out in mice to confirm its antidiarrheal effect. Acute toxicity study was performed to determine safe dose range before *in vivo* experiments.

**Results:**

In isolated rabbits’ jejunal preparations, Rm.Cr inhibited the spontaneous and high K^+^-induced contractions with respective EC_50_ values of 0.66 (0.44-0.97; n = 5) and 2.28 mg/mL (1.43-3.62; n = 5), like that of verapamil. This suggests the presence of calcium channel blocking (CCB) activity as a possible mode of action. The Ca^++^ channel blocking activity was further confirmed when pre-treatment of isolated jejunums with Rm.Cr (1-5 mg/mL) caused a rightward shift in the Ca^++^ concentration-response curves (CRCs), similar to verapamil. Rm.Cr was safe up to 2000 mg/kg for *in vivo* acute toxicity. Rm.Cr provided 55% and 80% protection from diarrhoea in respective doses of 100 mg/kg and 1000 mg/kg. These data indicates that the crude extract of *Rosa moschata* possesses Ca^++^ antagonist-like constituent(s), which explains its inhibitory effect on gut motility; a mechanism that underlies its antidiarrheal and antispasmodic activities.

**Conclusion:**

The study shows that the crude extract of fruits of *Rosa moschata* possesses antispasmodic effects mediated possibly through voltage gated Ca++ channel blockade, which provides sound pharmacological base to its medicinal use in gut spasms and diarrhoea, though additional mechanism(s) cannot be ruled out.

## Background

Plants have been used from a long while for the treatment of various diseases and 80,000 species out of 250,000 of higher plants are used medicinally [1]. The use of natural products are considered safe as compared to synthetic products, so the beliefs on using the synthetic products is decreasing compared to natural products [2]. Herbal medicines show potential uses in future because most of the plants, their activities and pharmacological activities have not being explored completely [3].

*Rosa moschata* belongs to a family called rosaceae [4]. There are more than 120 species of rose. Seven species of rosaceae are found in Malakand region [4, 5]. The local names are “zangley gulap”, “kurach”,“Qorach” in Pakistan and “kuja”,“Kojai”,“kunia” in India [4, 6–10]. It is small perennial climbing shrub and its flowers, leaves, fruits or whole plant is used medicinally. It is traditionally used in eyes’ disorders, diarrhoea, wounds healing, stomach disorders, delivery cases and in bilious affections [6, 8, 11–13]. It has also vermicidal properties [9]. Folklorically, the *Rosa moschata* is also used as laxative agent [14]. However, as laxative agent, no specific “part used” have been refernced thereby providing a space for resaerchers to explore. Its antioxidant property has been explored on scientific background [15].

The fatty acid composition of *Rosa moschata* has been determined by gas chromatography and then confirmed by the gas chromatography mass spectroscopy (GC-MS) which contains stearic acid, palmitic acid, oleic acid, margaric acid, linoleic acid and linolenic acid. The other isolated compounds of *Rosa moschata* are Vitamin A, C, E, flavonoids and essential oil [5, 16, 17]. Other species of the genius rosa like *Rosa damascena* have been reported to have spasmogenic (low concentration) and spasmolytic activity (in higher concentration) on rats’ ileum [18]. In addition, relaxant activity of *Rosa damascena* on guinea pig tracheal chains has been reported [19].

Due to its medicinal use in gut spasm and diarrhea, the current work focused to provide pharmacological basis for the medicinal use of *Rosa moschata* in gut spasms.

## Methods

### Plant materials and extraction of crude extract

Fresh fruits of *Rosa moschata* were collected from Malakand region and authenticated by Professor Dr. Jehandar Shah, ex vice chancellor and plant taxonomist, University of Malakand, Pakistan. A voucher specimen (RM-2103) has been deposited in the Department of Pharmacology, Khyber Medical University, Peshawar.

Plant materials, free of adulterants, were repeatedly extracted (3 times) with commercial grade methanol (80%) at room temperature and the combined extract was evaporated in rotary evaporator at 35-40°C to a semisolid mass, the crude extract of *Rosa moschata*. The extract (free of solvent) was solubilized in normal saline and distilled water for the *in-vivo* and *in-vitro* experiments, respectively.

### Drugs and standards

Analytical grade chemicals were used throughout these experiments. Acetylcholine was purchased from BDH, Poole, England, which was used for the maintenance of tissues at quiescent doses. Rest of the chemicals were of E Merck grade. Stock solutions of all the chemicals were made in distilled water and the dilutions were made fresh in normal saline on the days of experiments.

### Animals

BALB/C mice (weighing 25-30 g, hired from NIH, Islamabad) and local breed rabbits (weighing 1.5-2 kg) of either sex were housed at the animal house of the Institute of Basic Medical Sciences, Khyber Medical University, Peshawar, Pakistan under a controlled environment (23-25°C). The animals were kept in respective cages and were fasted overnight before starting the experiments. Advanced Study & Research Board and Ethical Board of Khyber Medical University approved the study protocols (ASRB000152/AA/IBMS/20/03/2014).

### Preliminary phytochemical screenings

Plant extracts was tested for various active principles i.e. Triterpenoids, Steroids, Glycosides, Saponins, Alkaloids, Flavonoids, Tannins, and Carbohydrate using different tests such as Liebermann Burchard test was used for steroids and triterpenoids, Keller Killiani and Bromine water test for Glycosides, Foam test for Saponins, Hager's test for Alkaloids, Ferric chloride test, Alkaline reagent test and Lead acetate solution test for Flavonoids, Gelatin test for Tannins, Biuret test for proteins and Benedict's test for carbohydrates described by Bhaddray, 2012 [20].

### Acute toxicity

Acute toxicity was performed as reported previously by Ali et al. 2013 [21]. Briefly, animals were divided in groups of 5 mice in each group. The test was performed in test doses of 1, 10, 1000 and 2000 mg/kg, given through intraperitoneal route. Another group of mice was administered normal saline which served as negative control. The mice were allowed food and water ad libitum during 24 hours test period. The animals were under regular observations for gross behavioral changes and mortality during the said period.

### Isolated tissue preparations

The isolated tissues experiments were performed in accordance with protocols established in our lab [21, 22]. Rabbits were fasted for 24 hours before the experiments with free access to water. Then rabbits were sacrificed by cervical dislocation, the abdomens were cut open and the jejunal portions were isolated. Preparations of about 2 cm long were mounted in 15 mL tissue baths containing Tyrode’s solution maintained at 37°C, constantly aerated with carbogen (a mixture of 5% carbon dioxide in oxygen). The composition of Tyrode’s, in mM, was: NaCl 136.9, KCl 2.7, NaHCO_3_ 11.9, MgCl_2_ 1.1, Glucose 5.6, NaH_2_PO_4_ 0.4, and CaCl_2_ 1.8 (pH 7.4). Tension of about 1.0 g of preload was applied to tissues and subsequently kept undisturbed for 30 min for stabilization. Upon stabilization of the isolated jejunal tissues, reproducible control responses of acetylcholine (0.3 μM) were obtained.

Under these experimental conditions, rabbits’ jejunums exhibited spontaneous contractions, allowing testing for possible relaxant (spasmolytic) activity directly without use of a spasmogen or an agonist.

### Calcium antagonist activity

To assess whether the spasmolytic activity of the test substances was mediated through voltage gated Ca++ channels blockade, high concentration of K+ (80 mM), as KCl, was used to depolarize the preparations [21, 22], which produced sustained contractions. Cumulative dosing of verapamil (standard) and extract of Rm.Cr was then added to isolated tissue baths to obtain concentration-dependent inhibitory responses. The relaxation of isolated jejunal tissues, pre-contracted with K+ was expressed as percent of the control pre-contractions.

Ca^++^ channel blocking activity of plant extract was confirmed when the tissues were first stabilized in normal Tyrode’s solution and normal Tyrode’s solution was replaced with Ca^++^ free Tyrode’s solution containing EDTA (0.1 mM) for 30 minutes. EDTA chelates the Ca^++^. This solution was then replaced with K^+^-rich and Ca^++^-free Tyrode's solution, having the following composition: NaCl 91.04, KCl 50, NaHCO_3_ 11.90, MgCl_2_ 1.05, glucose 5.55, NaH_2_PO_4_ 0.42 and EDTA 0.1 mM. Control concentration-response curves (CRCs) of Ca^++^ were obtained after an incubation period of 30 minutes. When the CRCs of Ca^++^ were found superimposable (usually after two cycles), the tissues were then pretreated with Rm.Cr for 60 minutes to test the possible Ca^++^ channel blocking effect. The CRCs of Ca^++^ were reconstructed in the presence of different concentrations of the plant extract and verapamil (standard).

### Data recording and Interpretation

Isotonic Transducer (MLT 0210/A Pan Lab) were used to record the intestinal recordings, connected with Power lab (Model No: 4/26 T) AD Instruments, Australia. Bridge Pod Amplifier connected with the Power lab was used for amplification of the intestinal responses.

Lab Chart 7 supplied with the power Lab was used to record and interpret the data.

### Effects on castor oil-induced diarrhea

Based on positive relaxant activity of Rm.Cr on gut mortality, the *in-vivo* antidiarrheal activity of the extract was carried out following the methods as described in our previous articles with slight modifications [23]. In this study, Balb C albino mice were kept on overnight fasting. Five groups of the mice were made and kept in five different steel cages containing four mice in each. Bottom of each cage was covered with blotting sheet. Normal saline (10 mL/kg, orally) was given to first group and labeled as the negative control. Rm.Cr was administered orally by intra-gastric feeding needle to the second, third and fourth group in doses of 100, 300 and 1000 mg/kg keeping in view the acute toxicity results. Loperamide (10 mg/kg p.o.) was given to the fifth group as positive control. 10 mL/kg of castor oil (p.o.) was given to all the groups after one hour post extract/test sample treatment. All the groups were then observed for the presence of diarrhoeal droppings in the individual cages for 4 hours after ingestion of the castor oil. Based on the number of dry and wet feces, percent protection against the castor oil-induced diarrhoea was calculated for each group.

### Data analysis

Data are expressed as mean ± standard error of the mean (SEM) and the median effective concentrations (EC_50_) values were given with 95% confidence intervals (CI) using Graph Pad Prism.

## Results and discussion

**Figure 1:**
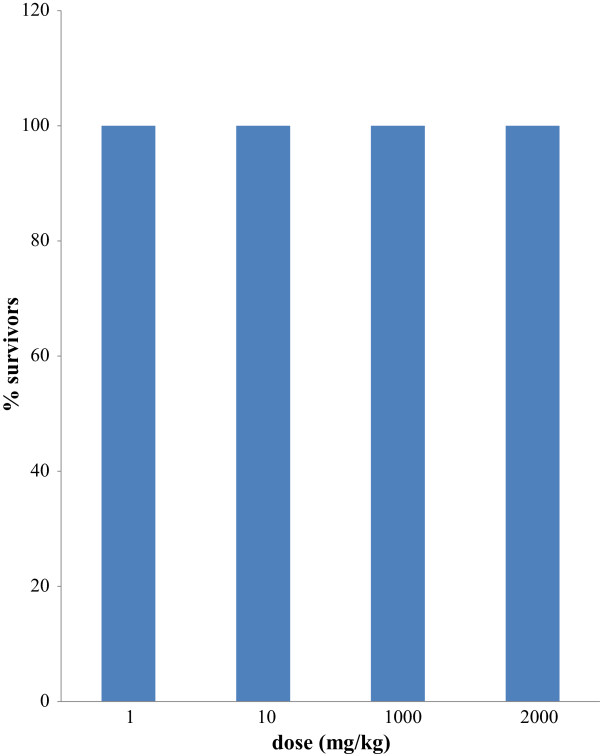
**Effect of acute treatment of** ***Rosa moschata*** **extract on % survivors of mice.**

Upon preliminary phytochemical screenings, *Rosa moschata* tested positive for presence of tannins, flavonoids, saponins, phenolics, proteins and terpenoids. It tested negative for alkaloids, quinones, sterols and glycosides (Table 1). Acute toxicity results proved that the extract is safe up to 2000 mg/kg. The percent survivors are expressed in Figure 1. This reflects good safety profile of the sample making it a possible candidate for the development of herbal products.

**Table 1:** **Phytochemical screenings of** ***Rosa moschata***

s. no	Phytochemicals	Remarks
1.	Alkaloids	-
2.	Flavonoids	+
3.	Tannins	+
4.	Saponins	+
6.	Carbohydrate	+
5.	Quinones	-
7.	Sterols	-
8.	Phenolics	+
9.	Proteins	+
10.	Terpenoids	+
11.	Glycosides	-

**Figure 2:**
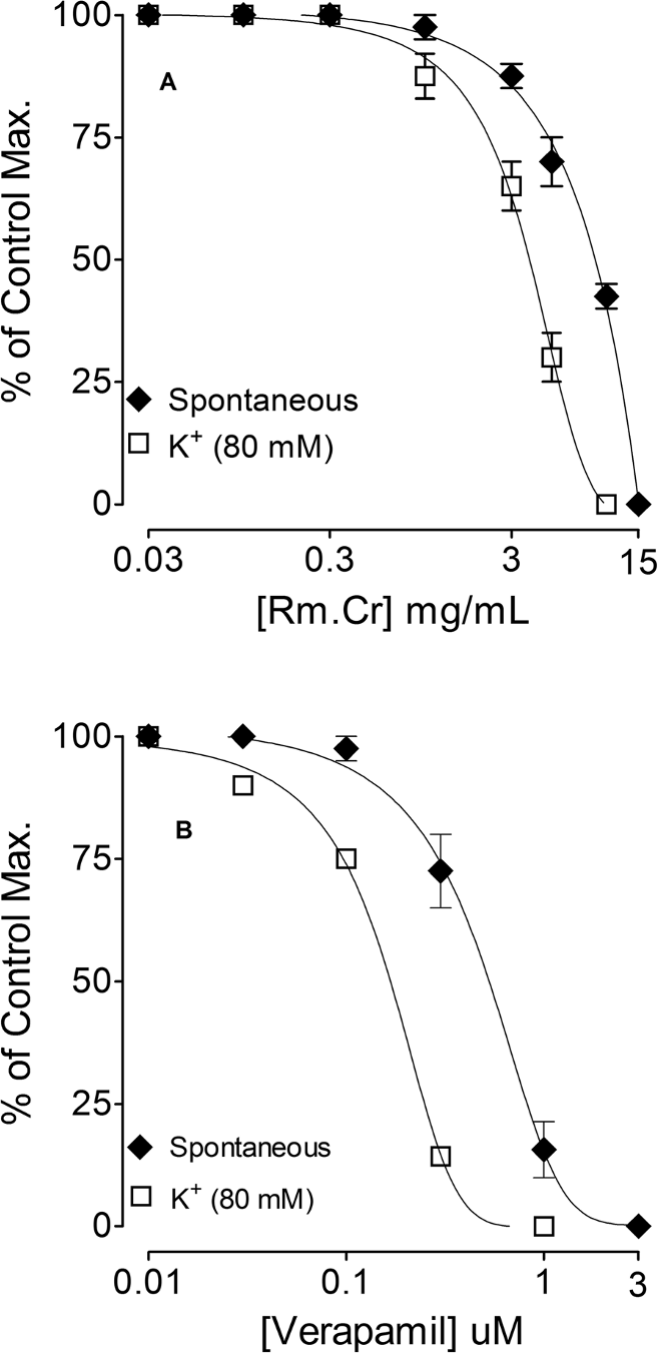
**Concentration-response curves of; (A) the crude extract of** ***Rosa moschata*** **(Rm.Cr), and (B) verapamil on spontaneous and high K** ^**+**^ **(80 mM)-induced contractions.** Values shown are mean ± SEM (n = 5).

**Figure 3:**
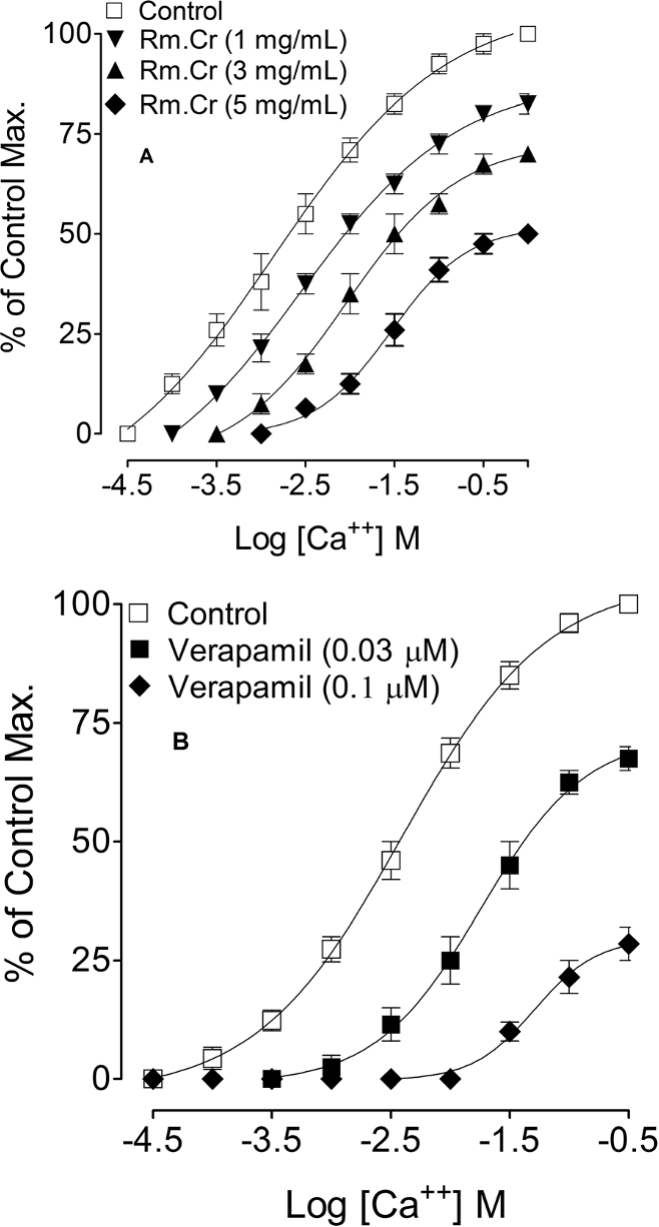
**Effect of different concentrations of Rm.Cr (A, n = 5) and verapamil (B, n = 7) on Ca++ concentration-response curves in isolated rabbits’ jejunal preparations.**

As there is no study available on the antispasmodic and antidiarrheal activity of this plant, therefore, this study was undertaken to provide the pharmacological basis for its medicinal use in hyperactive gut disorders and to investigate the mechanism of its spasmolytic effect. For this purpose this study was carried out on gut motility in isolated spontaneously contracting rabbits’ jejunums, where cumulative addition of crude extract of *Rosa moschata* caused concentration-dependent inhibition of the spontaneous and high K^+^ induced contractions, with EC_50_ values of 8.21 mg/mL (6.66 – 10.12) and 3.93 (3.39 - 4.55), respectively (Figure 2A). In similar pattern, verapamil, a standard Ca^++^ antagonist [22], relaxed the spontaneous and high K^+^ induced contractions, with EC_50_ values 0.51 mg/mL (0.43 – 0.60) and 0.16 mg/ml (0.13 – 0.21), respectively (Figure 2B). This shows smooth muscle relaxant (antispasmodic) activity may be mediated through calcium antagonistic effect as high K^+^ (>30 mM) is known to cause smooth muscle contractions through opening of voltage-dependent L-type Ca^++^ channels, thus allowing influx of extracellular Ca^++^ causing a contractile effect [24] and the substance which cause inhibition of high K + -induced contraction is considered to be an inhibitor of Ca^++^ influx [25]. Extract of the *Rosa maschata* relaxed the high K + -induced contractions, similar to that caused by verapamil (standard Ca^++^ antagonist [26] indicating its CCB action. The Ca^++^ antagonist effect of *Rosa moschata* was further confirmed when Rm.Cr dose dependently (1–5 mg/mL) shifted the Ca^++^ concentration response curves to the right (Figure 3A), like that caused by verapamil (Figure 3B). Ca^++^ antagonists have been shown to be beneficial in gut disorders resulting from hyperactivity such as diarrhoea and abdominal cramps [27]; hence the observed CCB effect justifies the medicinal use of *Rosa moschata* in such conditions. This observed Calcium channel blocking effect of the plant may be due to the presence of flavonoids, as evident from phytochemical screening, because the constituents of this class of compounds have been reported to have Calcium channel blocking activity [28, 29], however, contribution of other compound may not be ruled out. This is the first functional study on the gut motility with possible mode of action, carried out on the fruit of *Rosa moschata.*

**Figure 4:**
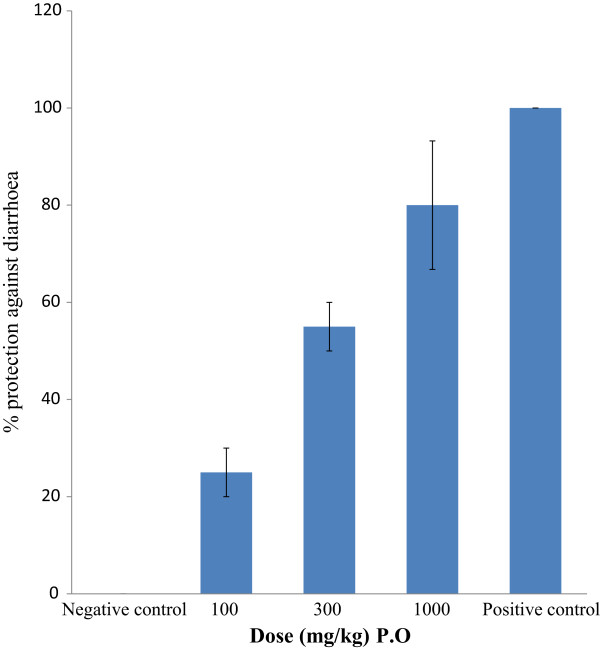
**Effects of the crude extract of the leaves of** ***Rosa moschata*** **and loperamide on castor oil-induced diarrhea in mice (mean** **±** **SD; n =3).**

Based on the relaxant effect of *Rosa moschata* on spontaneous and K^+^ induced contractions in isolated jejunal preparations, an *in vivo* model was used to test the extract for possible inhibitory effect on gut motility as for possible antidiarrhoeal activity. The crude extract provided protection from diarrhea in castor oil-induced diarrhea, similar to loperamide, a standard antidiarrheal agent [30]. Both extract and loperamide significantly inhibited (P < 0.05) the frequency of defecation as well as wetting of feces when compared with the untreated group (i.e. mice which only received castor oil but neither crude extract, nor loperamide). The percent protection provided by the crude extract was 25 ± 5, 55 ± 5 and 80 ± 13.2 at doses of 30, 100 and 1000 mg/kg respectively. Loperamide provided 100% protection at 10 mg/kg (Figure 4).

Hydrolysis of castor oil results in the formation of recinoleic acid [31], which produces changes in the transport of water and electrolytes resulting in a hyper secretory response and generation of a giant contraction of the intestine [32]. Thus, a potential antidiarrheal agent may exhibit its antidiarrheal effect by inhibiting either contraction of smooth muscles of the gut or electrolyte out flux [33].

## Conclusion

In summary, this study shows that the crude extract of *Rosa moschata* possesses antispasmodic and antidiarrheal effects, mediated possibly through Ca^++^ channel blockade, which provides sound pharmacological base to its medicinal use in diarrhoea and gut spasms, though additional mechanism(s) cannot be ruled out.

## Abbreviations

Rm.Cr: Crude methanolic extract of *Rosa moschata*
CCB: Calcium channel blocking
CRCs: Concentration-response curves.
